# A VLP-based vaccine targeting domain III of the West Nile virus E protein protects from lethal infection in mice

**DOI:** 10.1186/1743-422X-7-146

**Published:** 2010-07-06

**Authors:** Gunther Spohn, Gary T Jennings, Byron EE Martina, Iris Keller, Markus Beck, Paul Pumpens, Albert DME Osterhaus, Martin F Bachmann

**Affiliations:** 1 Cytos Biotechnology, Wagistrasse 25, 8952 Schlieren, Switzerland; 2 Erasmus MC, Department of Virology, P.O. Box 2040, 3000 CA Rotterdam, The Netherlands; 3 Latvian Biomedical Research and Study Centre, Ratsupites iela 1, Riga, LV 1067, Latvia; 4 AO Foundation, Clavadelerstrasse 8, 7270 Davos Platz, Switzerland; 5 ETH Zürich, Institute for Integrative Biology, Universitätstrasse 16, 8092 Zürich, Switzerland

## Abstract

**Background:**

Since its first appearance in the USA in 1999, West Nile virus (WNV) has spread in the Western hemisphere and continues to represent an important public health concern. In the absence of effective treatment, there is a medical need for the development of a safe and efficient vaccine. Live attenuated WNV vaccines have shown promise in preclinical and clinical studies but might carry inherent risks due to the possibility of reversion to more virulent forms. Subunit vaccines based on the large envelope (E) glycoprotein of WNV have therefore been explored as an alternative approach. Although these vaccines were shown to protect from disease in animal models, multiple injections and/or strong adjuvants were required to reach efficacy, underscoring the need for more immunogenic, yet safe DIII-based vaccines.

**Results:**

We produced a conjugate vaccine against WNV consisting of recombinantly expressed domain III (DIII) of the E glycoprotein chemically cross-linked to virus-like particles derived from the recently discovered bacteriophage AP205. In contrast to isolated DIII protein, which required three administrations to induce detectable antibody titers in mice, high titers of DIII-specific antibodies were induced after a single injection of the conjugate vaccine. These antibodies were able to neutralize the virus in vitro and provided partial protection from a challenge with a lethal dose of WNV. Three injections of the vaccine induced high titers of virus-neutralizing antibodies, and completely protected mice from WNV infection.

**Conclusions:**

The immunogenicity of DIII can be strongly enhanced by conjugation to virus-like particles of the bacteriophage AP205. The superior immunogenicity of the conjugate vaccine with respect to other DIII-based subunit vaccines, its anticipated favourable safety profile and low production costs highlight its potential as an efficacious and cost-effective prophylaxis against WNV.

## Background

West Nile virus (WNV) is a positive-stranded RNA flavivirus grouped within the Japanese encephalitis virus serocomplex. Transmitted primarily between birds via *Culex *mosquitoes, it occasionally infects humans, where it usually remains asymptomatic or causes a mild undifferentiated febrile illness called West Nile fever. Under certain conditions, mainly in immunocompromised or elderly individuals, and in individuals deficient in expression of the chemokine receptor CCR5, WNV infection can develop into severe, potentially life-threatening encephalitis [1-4]. In 2002, WNV was responsible for the largest outbreak of arthropod-borne encephalitis recorded in the USA, accounting for 2946 diagnosed cases and 284 deaths [5]. Since then the virus has been spreading throughout the USA, as well as Canada, Mexico and the Caribbean basin [6]. Isolated clinical cases have also been reported in recent years in Mediterranean countries, suggesting emergence of the virus in Western Europe [7,8]. In the absence of an effective treatment, there is a medical need for the development of a safe and efficient prophylactic vaccine against WNV.

A chimeric virus incorporating the envelope proteins of WNV into the infectious backbone of a yellow fever vaccine strain is currently being developed as a live-attenuated vaccine [9-11]. While immunogenic in humans, such a vaccine carries the inherent risk of reversion to a more virulent form, requiring stringent monitoring of the production process and careful safety assessment during clinical development. Alternative vaccination strategies are therefore focusing on recombinant subunit vaccines based on the large envelope glycoprotein (E) of WNV. The E protein is crucial for virus attachment and entry into host cells and is also the major antigen eliciting neutralizing antibody responses [12]. In particular a structurally distinct domain of the E protein (DIII) has been proposed as the receptor-binding domain [13]. Antibodies recognizing epitopes in this domain have been shown to neutralize the virus in vitro [14-19] and passive transfer of DIII-specific antibodies has been shown to protect mice from WNV challenge [19]. Subunit vaccines based on recombinantly expressed DIII have been tested in animal models and have proven effective in protecting from WNV infection [20-24]. However, multiple injections and/or strong adjuvants were needed to induce neutralizing antibody responses, indicating that isolated DIII is poorly immunogenic.

We have previously shown that by displaying antigens in a repetitive and highly ordered fashion on the surface of virus-like particles (VLPs) derived from the bacteriophage Qβ, specific B cells can be efficiently activated and rapid and robust antibody responses can be induced [25-28]. Here we describe the production of a conjugate vaccine based on recombinant DIII covalently linked to VLPs derived from the recently discovered bacteriophage AP205. A single injection of the conjugate vaccine was sufficient to induce virus-neutralizing antibodies and provide significant protection from WNV challenge, demonstrating its superior immunogenicity over previously described DIII-based vaccines and highlighting its potential as an efficient and safe prophylaxis against WNV.

## Results

### Production of the AP205 VLP Carrier

**Figure 1:**
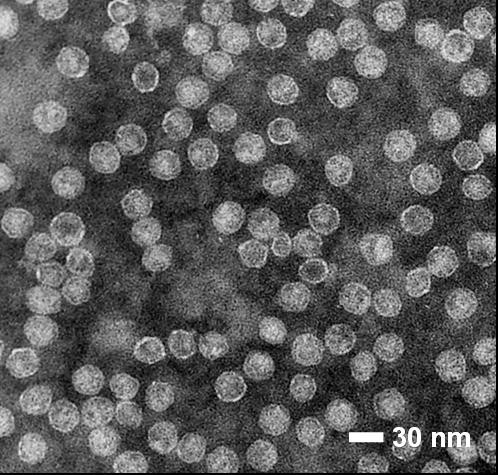
**Electron micrograph of purified AP205 VLPs**. Purified AP205 VLPs were adsorbed onto carbon-Formvar-coated grids, stained with 2% phosphotungstic acid and subjected to transmission electron microscopy.

The bacteriophage AP205 has first been isolated from *Acinetobacter *spp. and belongs to the *Leviviridae *family, a group of ssRNA phages which infects a wide range of Gram-negative bacteria. Its positive-stranded RNA genome comprises three large open reading frames, which encode the maturation, coat and replicase proteins [29]. The icosahedral T = 3 phage particles are 29 nm in size and contain the genomic RNA in a densely packaged fashion [30]. In analogy to the coat proteins of the related phages Qβ [31] and MS2 [32], the AP205 coat protein self-assembles into virus-like particles upon recombinant expression in *E. coli *[33]. We purified AP205 VLPs from lysates of *E. coli *overexpressing the coat protein by sequential anion exchange and mixed-mode cation/anion exchange chromatography. Figure 1 shows that AP205 VLPs purified in this way were 25-30 nm in size and strongly resembled in their appearance the wild type particles [29]. Spectrophotometric analysis and agarose gel electrophoresis indicated that AP205 VLPs contained approximately 25-30 μg of host cell RNA per 100 μg of coat protein (not shown).

### Production of the DIII-C-AP205 Conjugate Vaccine

**Figure 2:**
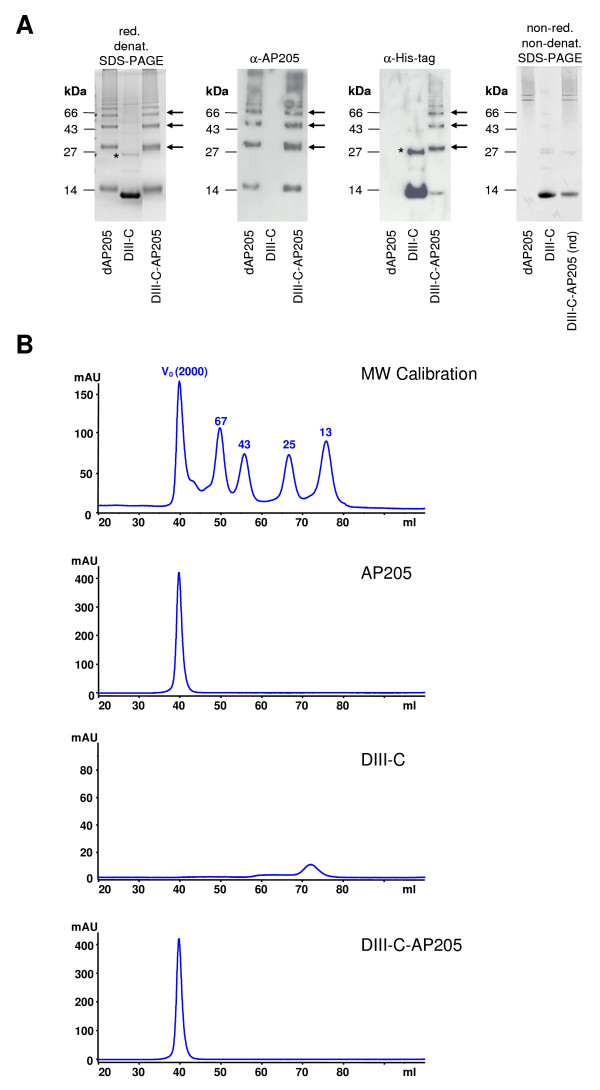
**Production and characterization of the DIII-C-AP205 conjugate vaccine**. **A **Derivatized AP205 (dAP205), DIII-C, and the dialysed conjugate vaccine (DIII-C-AP205) were analyzed by reducing, denaturing SDS-PAGE (*left panel*). Corresponding amounts of derivatized AP205, DIII-C, and of the non-dialysed (nd) conjugate vaccine were also analysed by non-reducing, non-denaturing SDS-PAGE (*right panel*). For identification of the coupling bands, proteins were separated on reducing, denaturing SDS-PAGE, blotted on nitrocellulose and detected with AP205- and His-tag- specific antibodies (*middle panels*). Bands corresponding to AP205-crosslinked DIII-C are indicated by arrows. The 27 kDa band which is visible in the SDS-PAGE and in the His-tag-specific Western Blot corresponds to dimeric DIII-C (*). **B **Size exclusion chromatography. A superdex 75 column was calibrated with a molecular weight (MW) calibration kit and then loaded sequentially with the indicated proteins. AP205 VLPs and the conjugate vaccine DIII-C-AP205 elute in the void volume of the column (V_0_, 40 ml) while purified DIII-C elutes at 72.4 ml.

Domain III of the WNV E protein was engineered to comprise a hexahistidine tag and a cysteine-containing linker at its C-terminus (DIII-C). Upon expression in *E. coli *the protein formed insoluble inclusion bodies. DIII-C was solubilized in urea, purified under denaturing conditions by affinity chromatography and refolded into its native form by stepwise dialysis. A heterobifunctional cross-linker was used to covalently conjugate DIII-C via the introduced C-terminal cysteine residue to lysine residues on the surface of AP205 VLPs. The isolated vaccine components and the conjugate vaccine (DIII-C-AP205) were analysed by SDS-PAGE and Western Blot, as well as size exclusion chromatography, with results shown in Figure 2. Recombinant DIII-C migrated as a distinct 13.5 kDa band in reducing, denaturing SDS-PAGE (Figure 2A, left panel), consistent with its predicted molecular weight. Size exclusion analysis of the protein showed a single peak with an elution volume corresponding to an apparent molecular weight of approximately 16 kDa, indicating that DIII-C was indeed a folded monomer in solution (Figure 2B). Analysis of the conjugate vaccine DIII-C-AP205 showed the presence of several high molecular weight bands in reducing, denaturing SDS-PAGE, which reacted with both AP205- and His-tag specific antisera, demonstrating the successful crosslinking of DIII-C to the AP205 VLPs (Figure 2A, middle panels). Similar to AP205 VLPs, the DIII-C-AP205 conjugate vaccine eluted in the void volume of the size exclusion column, confirming its virus-like particle assembly state (Figure 2B). In order to estimate the number of DIII-C molecules displayed by each VLP in the conjugate vaccine, the corresponding amounts of DIII-C used in the coupling reaction and of the non-dialysed DIII-C-AP205 conjugate vaccine were loaded side by side on a non-reducing, non-denaturing SDS polyacrylamide gel (Figure 2A, right panel). Under these conditions, DIII-C still migrates as a 13.5 kDa monomer while the conjugate vaccine migrates as a high molecular weight complex. Densitometric analysis showed a 55% decrease in the amount of free DIII-C after coupling to AP205. Assuming that the decrease in free DIII-C is due to coupling to AP205 subunits and taking into account the molar ratio of VLP subunits and DIII-C molecules used in the coupling reaction it could be calculated that an average 27% of AP205 subunits had been cross-linked to DIII-C molecules. As each AP205 VLP is composed of 180 subunits, it can be estimated that an average of 50 DIII-C molecules are displayed per vaccine particle.

### Immunogenicity of DIII-C-AP205 in Mice

**Figure 3:**
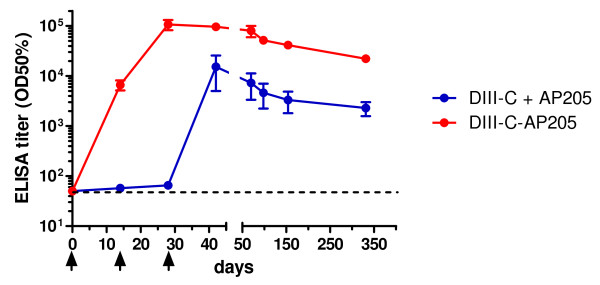
**Immunogenicity of DIII-C-AP205**. Groups of female BALB/c mice (n = 4) were immunized subcutaneously three times (days 0, 14, and 28, arrows) with either 50 μg of DIII-C-AP205 or a mixture of the corresponding amounts of free DIII-C protein (13.6 μg) and free AP205 VLPs (36.4 μg) in the absence of adjuvants. DIII-C-specific IgG antibody titers were measured at the indicated time points. The dashed line indicates the detection limit. Shown are group means ± SEM.

Groups of mice were immunized subcutaneously either with the DIII-C-AP205 conjugate vaccine or a mixture of the corresponding amounts of non-conjugated AP205 carrier and free DIII-C protein in the absence of any additional adjuvant. Figure 3 shows that one or two injections of the non-conjugated AP205/DIII-C mixture did not result in any detectable DIII-specific IgG antibodies, confirming the notion that isolated DIII is poorly immunogenic. A measurable IgG antibody response could be induced only after a third injection (ELISA titer of 15,300 on day 42). In contrast, DIII-specific IgG titers were measured after only a single administration of the DIII-C-AP205 conjugate vaccine (6,600 on day 14). A second vaccine injection boosted the specific antibodies to a titer of 106,900 (day 28), while a third injection did not lead to a further increase. In the absence of additional injections, antibody titers slowly declined with an approximate half life of two months.

### Vaccination with DIII-C-AP205 Induces Neutralizing Antibodies and Protects from Lethal WNV Infection

**Figure 4:**
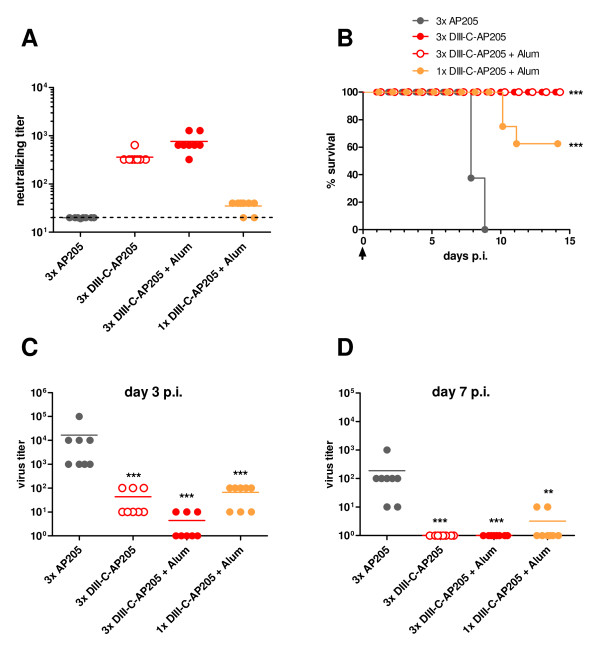
**Immunization with DIII-C-AP205 protects from lethal WNV infection**. **A **Induction of neutralizing antibodies. Groups of female C57BL/6 mice (n = 8) were immunized subcutaneously on days 0, 14 and 28 with 50 μg DIII-C-AP205 either in the absence or presence of Alum as adjuvant or with 50 μg AP205 VLPs in the absence of adjuvant. A fourth group was immunized once with 50 μg DIII-C-AP205 in Alum on day 28. Virus-neutralizing titers of individual sera were measured on day 42. The dashed line indicates the detection limit. Shown are individual titers and group means. **B **Protection from WNV challenge. Two weeks after the last vaccine injection mice were challenged with a lethal dose of WNV (arrow). Statistical significance of differences in survival curves was calculated by log-rank test using GraphPad-Prism (***p < 0.001 vs. 3xAP205 control). p.i.= post infection **C **and **D **Viral titers were determined 3 and 7 days post infection (p.i.) in blood of infected animals. Shown are individual titers and group means. The Mann-Whitney test was used to assess statistical significance (**p < 0.01, ***p < 0.001 vs. 3xAP205 control).

Groups of mice were immunized with DIII-C-AP205 three times (days 0, 14, 28) either in the absence or in the presence of Alum as adjuvant. One group was immunized only once (day 28) in the presence of Alum. The neutralizing capacity of the induced antibodies was determined two weeks after the last injection (day 42). Figure 4A shows that three injections of DIII-C-AP205 in the absence or presence of adjuvant resulted in average neutralizing titers of 360 and 760, respectively. Neutralizing ability was also detected in sera from 6 of the 8 mice that that received a single injection of vaccine. All vaccinated mice and a group of control mice, that had been immunized with the AP205 carrier alone, were then challenged with a lethal dose of West Nile virus strain NY99. Figure 4B shows that all mice that had received three injections of DIII-C-AP205 either in the presence or absence of Alum survived the viral challenge. In contrast, all control mice immunized with AP205 succumbed to the infection. Interestingly, 5 out of 8 mice that had received a single injection of DIII-C-AP205 in Alum also survived the challenge with WNV. This survival rate is in the range of the ones achieved after single doses of different live attenuated WNV vaccines in mice [9,34,35]. Vaccination with DIII-C-AP205 resulted in a marked reduction of viremia as measured by real time PCR in blood 3 and 7 days after infection (Figures 4C and 4D, respectively). All mice that had received three injections of DIII-C-AP205, as well as 6 out of 8 mice that had received a single injection had cleared the virus by day 7; the 2 remaining mice of this group had reduced viral titers as compared to the average viral load of AP205-immunized animals at this time point.

## Discussion

Domain III of the envelope protein of WNV is a major target of virus-neutralizing antibody responses and has been identified as a promising candidate antigen for the development of recombinant subunit vaccines [36]. In this study we produced a highly immunogenic and efficacious WNV vaccine consisting of recombinant domain III chemically cross-linked to virus-like particles of the bacteriophage AP205. The conjugate vaccine produced high DIII-specific antibody titers in mice, which were able to efficiently inhibit viral replication both *in vitro *and *in vivo*. In contrast to other experimental vaccines based on recombinant DIII and comprising different adjuvants [20-23], a single injection in the presence of Alum was sufficient to induce neutralizing antibody responses and confer partial protection from WNV challenge (Figure 4). Interestingly, the neutralizing titers observed (approximately 30-40) were in the range of those induced by a single injection of live WNV vaccines such as a chimeric attenuated flavivirus vaccine [9,35] or a recombinant attenuated influenza strain expressing the WNV E protein [34]. Multiple injections either in the presence or absence of Alum as adjuvant yielded sustained high titers of DIII-specific antibodies, which efficiently neutralized the virus. The reason for the superior immunogenicity of the DIII-C-AP205 conjugate vaccine most likely resides in its virus-like connotations. It has been shown that highly ordered and repetitive antigen arrays can cause an efficient cross-linking of BCRs on specific B cells and induce a rapid and sustained antibody response [37]. As domain III is presented to the immune system in an oriented and densely packaged fashion on the surface of the AP205 VLP, it is likely that DIII-specific B cells are promptly and efficiently activated to produce specific IgG antibodies. The particulate nature of the VLP vaccine furthermore ensures a preferential uptake by antigen-presenting cells such as dendritic cells, and thereby an efficient presentation of DIII- as well as AP205-derived epitopes on MHC class II for the priming of specific T_H _cells. Activation of antigen-presenting cells is also enhanced by bacterial RNA, which is spontaneously packaged into the VLP carrier during the recombinant expression and assembly process. Upon uptake by B cells and APCs, the RNA is co-delivered with the AP205 particle to the endosomal compartment, where it can activate TLR3 or TLR7/8 (for review see [38,39]).

In addition to its good immunogenicity, the DIII-C-AP205 vaccine is also expected to be safe and well tolerated. In contrast to live vaccines based on attenuated viruses, which inevitably carry the risk of genetic recombination and mutation into a more virulent form, DIII-C-AP205 is based on non-replicating virus-like particles derived from a bacteriophage, which are unable to infect mammalian cells. Moreover, both the VLP carrier and the antigen components of the vaccine can be produced in large amounts in bacterial expression systems and purified with relatively simple biochemical methods, suggesting that large scale production of the conjugate vaccine can be achieved in a cost-effective manner. A highly immunogenic, yet safe and affordable WNV vaccine would be attractive for veterinary prophylaxis and might also be used in elderly or immunocompromised individuals in high-risk areas. The high immunogenicity of the VLP vaccine might also offer the potential of inducing cross-protection against related flaviviruses such as Japanese encephalitis virus or dengue virus. That cross-protection may occur in principle has been shown by immunization of mice with recombinant domain III of the WNV E protein [21]. The increased immunogenicity of the domain III by conjugation to the VLP carrier may therefore be sufficient to confer cross-protective immunity.

## Conclusions

In the present study we show that the immunogenicity of DIII of the WNV E protein can be strongly enhanced by conjugation to virus-like particles of the bacteriophage AP205. In contrast to other vaccination approaches based on recombinant DIII, which require multiple injections and/or strong adjuvants for the induction of neutralizing antibodies, a single injection of the conjugate DIII-C-AP205 vaccine in Alum was sufficient to induce a significant amount of virus-neutralizing antibodies in mice. Three injections of the vaccine completely protected mice from a lethal WNV challenge, even when given in the absence of any adjuvant. The relatively low production costs of the DIII-C-AP205 vaccine, its superior immunogenicity with respect to other DIII-based approaches and its anticipated good safety profile make it an attractive candidate for WNV prophylaxis both in humans and in veterinary applications.

## Methods

### Expression and Purification of AP205 Virus-like Particles

Cleared bacterial lysates containing the recombinantly expressed coat protein of AP205 were dialysed against AEX loading buffer (20 mM NaH_2_PO_4 _pH 7.2) and loaded on a Fractogel™ TMAE column (Merck). After removal of host cell proteins by a salt wash with 333 mM NaCl, viral capsids were eluted with 600 mM NaCl and dialyzed against HAp loading buffer (5 mM NaH_2_PO_4_, 100 mM NaCl, pH 6.8). Capsids were bound to a hydroxyapatite column (Macro-prep ceramic hydroxyapatite type II, Biorad) and eluted with 60 mM NaH_2_PO_4_, 220 mM NaCl, pH 6.8, resulting in depletion of bacterial LPS.

### Expression and Purification of Recombinant Domain III of the E Glycoprotein of WNV

A DNA fragment encoding domain III of the glycoprotein E of WNV NY99 was amplified from plasmid pTRHis2A-WNV-E [21] with the oligonucleotide pair WNV1/WNV2 (5'-ATATAT**CATATG**GAAAAATTGCAGTTGAAGG-3'; 5'-ATATAT**CTCGAG**TTTGCCAATGCTGCTTCCAG-3', *Nde*I and *Xho*I restriction sites are in bold) and cloned into the expression vector pET42T [40]. The resulting plasmid encoded a fusion protein consisting of domain III of the WNV E protein (corresponding to amino acids 582-696 of the WNV polyprotein precursor), a hexahistidine tag, and a short C-terminal, cysteine containing linker (DIII-C). *E. coli *BL21 DE3 cells were transformed with this plasmid, and protein expression was induced in a logarithmic phase culture by addition of isopropyl-β-D-thiogalactopyranoside to a final concentration of 1 mM. After overnight growth bacteria were harvested by centrifugation, resuspended in 50 mM NaH_2_PO_4_, 150 mM NaCl, 10 mM MgCl_2_, 0.25% Triton X-100, pH 7.2, and lysed by sonication. Nucleic acids were digested by 1 h incubation at room temperature with 1500 U Benzonase (Sigma-Aldrich), and inclusion bodies containing recombinant DIII-C were harvested by centrifugation. After three washes with 100 mM Tris-Cl, 5 mM EDTA, 5 mM DTT, 2% Triton X-100, pH 7.0, inclusion bodies were solubilized in 8 M urea, 100 mM Tris-Cl, 100 mM DTT, pH 8.0, and loaded on a Ni-NTA column (Qiagen), which had been previously equilibrated with 8 M Urea, 100 mM NaH_2_PO_4_, 10 mM Tris-Cl, 2 mM β-mercaptoethanol, pH 8.0. Bound DIII-C was eluted with 8 M urea, 100 mM NaH_2_PO_4_, 10 mM Tris, 2 mM β-Mercaptoethanol pH 4.5, and dialysed against 2 M urea, 50 mM NaH_2_PO_4_, 0.5 M arginine, 0.5 mM oxidized glutathione, 5 mM reduced glutathione, 10% glycerol, pH 8.5. DIII-C was then refolded by stepwise dialysis against 50 mM NaH_2_PO_4_, 0.5 M arginine, 0.5 mM oxidized glutathione, 5 mM reduced glutathione, 10% glycerol, pH 8.5, and against 50 mM NaH_2_PO_4_, 10% glycerol, pH 8.5.

### Chemical Cross-linking of Recombinant DIII-C to AP205 Virus-like Particles

AP205 VLPs (in PBS, pH 7.2) were first reacted for 1 h at room temperature with a 2.5 fold molar excess of the heterobifunctional cross-linker succinimidyl-6-(β-maleimidopropionamido)hexanoate (Pierce). Free cross-linker was removed by dialysis against PBS, pH 7.2. Recombinant DIII-C was incubated for 1 h at room temperature with an equimolar amount of tri(2-carboxyethyl)phosphine-hydrochloride. Under these mildly reducing conditions the cysteine residue contained in the linker is reduced, while the internal disulfide bridge of DIII-C remains intact. The reduced protein was then mixed with the derivatized AP205 VLPs at a molar ratio of 1 DIII-C monomer per 2 AP205 monomers and incubated over night at 17°C to allow cross-linking. Free DIII-C was removed by extensive dialysis against PBS pH 7.2 using cellulose ester membranes with a cut-off of 100 kDa (Spectrum Laboratories). The conjugate vaccine was analyzed by SDS-PAGE followed by Coomassie Blue staining or by Western Blot using AP205- and His-tag- specific antisera. The molecular masses of the DIII-C and AP205 monomers are similar; 13.5 kDa and 14.0 kDa, respectively. The coupling product comprising one AP205 monomer covalently conjugated to one DIII-C monomer co-migrates with the AP205 dimer band. Hence the coupling efficiency could not simply be calculated by densitometry of protein bands on a reducing SDS-PAGE stained with Coomassie Blue. Instead the conjugate vaccine was loaded on a non-reducing non-denaturing SDS-PAGE side by side with the corresponding amount of free DIII-C, which had been used in the cross-linking reaction. By comparing the intensities of the DIII-C monomers before and after cross-linking to AP205 by densitometry, the amount of DIII-C coupled to the AP205 carrier could then be quantified.

### Analysis of DIII-C, AP205 and DIII-C-AP205 by Size Exclusion Chromatography

A superdex 75 column (GE Healthcare) was calibrated with a mixture of Dextran Blue (~2000 kDa), BSA (67 kDa), Ovalbumin (43 kDa), Chymotrypsinogen (25 kDa), and RNase A (14 kDa). AP205 VLPs, purified DIII-C protein and the conjugate vaccine DIII-C-AP205 were then sequentially analysed on the same column. The apparent molecular weight of DIII-C was calculated from a standard curve obtained by plotting the logarithm of the molecular weights of the protein standards against their partition coefficients.

### Immunogenicity of DIII-C-AP205

Female BALB/c mice (8 weeks of age) were purchased from Charles River Laboratories. DIII-C-AP205 vaccine or the mixture of the non-conjugated vaccine components AP205 and recombinant DIII-C were diluted in PBS to 200 μl and injected subcutaneously (100 μl on two ventral sites) in the absence of additional adjuvants. Sera from immunized mice were serially diluted in PBS containing 0.05% Tween-20, 2% BSA, and applied to ELISA plates (Nunc) that had been coated with 1 μg/ml recombinant DIII-C protein. Reactivity of serum antibodies with the target protein was determined using a HRP-conjugated goat anti-mouse IgG secondary antibody (Jackson ImmunoResearch Laboratories) at a dilution of 1:1000 in PBS/0.05% Tween-20/2% BSA. After development with 1,2-phenylenediamine dihydrochloride (0.4 mg/mL in 0.066 M Na_2_HPO_4_, 0.035 M citric acid, 0.01% H_2_O_2_, pH 5.0) the optical density at 450 nm (OD_450 nm_) was determined using an ELISA reader (Biorad). Titers were expressed as the reciprocal of those serum dilutions that lead to half-maximal OD_450 nm _(OD50%).

### WNV Challenge

Female C57BL/6 mice (6 weeks of age) were purchased from Harlan and allowed to acclimate to the facility for one week before experiments were performed. Experiments were approved by the animal ethics committee of the Erasmus MC Rotterdam, The Netherlands. Mice were immunized as indicated in the legend of Figure 4 and challenged two weeks after the last immunization by an intraperitoneal injection of a lethal dose of WNV-NY99 (1 × 10^6 ^TCID_50_). After the challenge, mice were maintained in isolation cages and observed daily for illness and death for a period of 14 days. Blood was collected on days 3 and 7 after infection and viral titers were determined by real-time PCR. The quantity of viral RNA was measured with a one-step RT-PCR TaqMan protocol and ABI PRISM 7500 detection instrument (EZ-kit, Applied Biosystems). The primers and probe used for WNV RNA quantification were: forward primer 5'-TCACTGTCAACCCTTTTGTTTC-3'; reverse primer 5'-AAGGGTGGTTCCAATTCAATC-3'; probe 5'-CCACGGCCAACGCTAAGGTCC-3'. Serial dilutions of WNV stock were used as standard, and results were expressed as TCID_50 _equivalents per gram of brain tissue. For determination of neutralizing antibody titers serial two-fold dilutions of immune sera were incubated with 100 TCID_50 _of WNV strain NY99. Virus-neutralizing titers were expressed as the reciprocal of the highest dilution that still resulted in 100% suppression of the cytopathic effects on Vero E6 cells [21].

## Competing interests

ADMEO is a part-time employee (CSO) of Viroclinics B.V. (for details go to http://www.erasmusmc.nl). The other authors declare that they have no competing interests.

## Authors' contributions

GS designed the experiments on vaccine production and immunogenicity testing, performed experiments on vaccine analytics, coordinated the study and drafted the manuscript. GTJ conceived and designed the project and helped to draft the manuscript. BEEM designed and performed the WNV infection experiments, helped to coordinate the study and to draft the manuscript. IK performed immunization and ELISA experiments. MB purified recombinant DIII-C, and produced the conjugate vaccine. PP performed the electron microscopy experiments. ADMEO and MFB conceived and designed the project. All authors read and approved the final manuscript.

## References

[B1] Campbell GL Marfin AA Lanciotti RS Gubler DJ West Nile virus Lancet Infect Dis 2002 2 519 529 10.1016/S1473-3099(02)00368-7 12206968

[B2] Hayes EB Sejvar JJ Zaki SR Lanciotti RS Bode AV Campbell GL Virology, pathology, and clinical manifestations of West Nile virus disease Emerg Infect Dis 2005 11 1174 1179 16102303 10.3201/eid1108.050289b PMC3320472

[B3] Glass WG McDermott DH Lim JK Lekhong S Yu SF Frank WA Pape J Cheshier RC Murphy PM CCR5 deficiency increases risk of symptomatic West Nile virus infection J Exp Med 2006 203 35 40 10.1084/jem.20051970 16418398 PMC2118086

[B4] Lim JK Louie CY Glaser C Jean C Johnson B Johnson H McDermott DH Murphy PM Genetic deficiency of chemokine receptor CCR5 is a strong risk factor for symptomatic West Nile virus infection: a meta-analysis of 4 cohorts in the US epidemic J Infect Dis 2008 197 262 265 10.1086/524691 18179388

[B5] O'Leary DR Marfin AA Montgomery SP Kipp AM Lehman JA Biggerstaff BJ Elko VL Collins PD Jones JE Campbell GL The epidemic of West Nile virus in the United States, 2002 Vector Borne Zoonotic Dis 2004 4 61 70 10.1089/153036604773083004 15018774

[B6] Gubler DJ The continuing spread of West Nile virus in the western hemisphere Clin Infect Dis 2007 45 1039 1046 10.1086/521911 17879923

[B7] Kaptoul D Viladrich PF Domingo C Niubo J Martinez-Yelamos S De Ory F Tenorio A West Nile virus in Spain: report of the first diagnosed case (in Spain) in a human with aseptic meningitis Scand J Infect Dis 2007 39 70 71 10.1080/00365540600740553 17366016

[B8] Rossini G Cavrini F Pierro A Macini P Finarelli A Po C Peroni G Di Caro A Capobianchi M Nicoletti L First human case of West Nile virus neuroinvasive infection in Italy, September 2008 - case report Euro Surveill 2008 13 18926106 10.2807/ese.13.41.19002-en

[B9] Arroyo J Miller C Catalan J Myers GA Ratterree MS Trent DW Monath TP ChimeriVax-West Nile virus live-attenuated vaccine: preclinical evaluation of safety, immunogenicity, and efficacy J Virol 2004 78 12497 12507 10.1128/JVI.78.22.12497-12507.2004 15507637 PMC525070

[B10] Monath TP Liu J Kanesa-Thasan N Myers GA Nichols R Deary A McCarthy K Johnson C Ermak T Shin S A live, attenuated recombinant West Nile virus vaccine Proc Natl Acad Sci USA 2006 103 6694 6699 10.1073/pnas.0601932103 16617103 PMC1436023

[B11] Guy B Guirakhoo F Barban V Higgs S Monath TP Lang J Preclinical and clinical development of YFV 17D-based chimeric vaccines against dengue, West Nile and Japanese encephalitis viruses Vaccine 28 632 649 10.1016/j.vaccine.2009.09.098 19808029

[B12] Roehrig JT Antigenic structure of flavivirus proteins Adv Virus Res 2003 59 141 175 full_text 14696329 10.1016/s0065-3527(03)59005-4

[B13] Lee JW Chu JJ Ng ML Quantifying the specific binding between West Nile virus envelope domain III protein and the cellular receptor alphaVbeta3 integrin J Biol Chem 2006 281 1352 1360 10.1074/jbc.M506614200 16275649

[B14] Beasley DW Barrett AD Identification of neutralizing epitopes within structural domain III of the West Nile virus envelope protein J Virol 2002 76 13097 13100 10.1128/JVI.76.24.13097-13100.2002 12438639 PMC136710

[B15] Volk DE Beasley DW Kallick DA Holbrook MR Barrett AD Gorenstein DG Solution structure and antibody binding studies of the envelope protein domain III from the New York strain of West Nile virus J Biol Chem 2004 279 38755 38761 10.1074/jbc.M402385200 15190071

[B16] Nybakken GE Oliphant T Johnson S Burke S Diamond MS Fremont DH Structural basis of West Nile virus neutralization by a therapeutic antibody Nature 2005 437 764 769 10.1038/nature03956 16193056 PMC7095628

[B17] Choi KS Nah JJ Ko YJ Kim YJ Joo YS The DE loop of the domain III of the envelope protein appears to be associated with West Nile virus neutralization Virus res 2007 123 216 218 10.1016/j.virusres.2006.09.002 17027114

[B18] Sanchez MD Pierson TC McAllister D Hanna SL Puffer BA Valentine LE Murtadha MM Hoxie JA Doms RW Characterization of neutralizing antibodies to West Nile virus Virology 2005 336 70 82 10.1016/j.virol.2005.02.020 15866072

[B19] Oliphant T Engle M Nybakken GE Doane C Johnson S Huang L Gorlatov S Mehlhop E Marri A Chung KM Development of a humanized monoclonal antibody with therapeutic potential against West Nile virus Nat Medicine 2005 11 522 530 10.1038/nm1240 PMC1458527 15852016

[B20] Wang T Anderson JF Magnarelli LA Wong SJ Koski RA Fikrig E Immunization of mice against West Nile virus with recombinant envelope protein J Immunol 2001 167 5273 5277 11673542 10.4049/jimmunol.167.9.5273

[B21] Martina BE Koraka P van den Doel P van Amerongen G Rimmelzwaan GF Osterhaus AD Immunization with West Nile virus envelope domain III protects mice against lethal infection with homologous and heterologous virus Vaccine 2008 26 153 157 10.1016/j.vaccine.2007.10.055 18069096 PMC7127062

[B22] Ledizet M Kar K Foellmer HG Wang T Bushmich SL Anderson JF Fikrig E Koski RA A recombinant envelope protein vaccine against West Nile virus Vaccine 2005 23 3915 3924 10.1016/j.vaccine.2005.03.006 15917113

[B23] Chu JH Chiang CC Ng ML Immunization of flavivirus West Nile recombinant envelope domain III protein induced specific immune response and protection against West Nile virus infection J Immunol 2007 178 2699 2705 17312111 10.4049/jimmunol.178.5.2699

[B24] McDonald WF Huleatt JW Foellmer HG Hewitt D Tang J Desai P Price A Jacobs A Takahashi VN Huang Y A West Nile virus recombinant protein vaccine that coactivates innate and adaptive immunity J Infect Dis 2007 195 1607 1617 10.1086/517613 17471430

[B25] Spohn G Guler R Johansen P Keller I Jacobs M Beck M Rohner F Bauer M Dietmeier K Kundig TM A virus-like particle-based vaccine selectively targeting soluble TNF-alpha protects from arthritis without inducing reactivation of latent tuberculosis J Immunol 2007 178 7450 7457 17513796 10.4049/jimmunol.178.11.7450

[B26] Spohn G Keller I Beck M Grest P Jennings GT Bachmann MF Active immunization with IL-1 displayed on virus-like particles protects from autoimmune arthritis Eur J Immunol 2008 38 877 887 10.1002/eji.200737989 18253928

[B27] Spohn G Schwarz K Maurer P Illges H Rajasekaran N Choi Y Jennings GT Bachmann MF Protection against osteoporosis by active immunization with TRANCE/RANKL displayed on virus-like particles J Immunol 2005 175 6211 6218 16237119 10.4049/jimmunol.175.9.6211

[B28] Rohn TA Jennings GT Hernandez M Grest P Beck M Zou Y Kopf M Bachmann MF Vaccination against IL-17 suppresses autoimmune arthritis and encephalomyelitis Eur J Immunol 2006 36 2857 2867 10.1002/eji.200636658 17048275

[B29] Klovins J Overbeek GP van den Worm SH Ackermann HW van Duin J Nucleotide sequence of a ssRNA phage from Acinetobacter: kinship to coliphages J Gen Virol 2002 83 1523 1533 12029168 10.1099/0022-1317-83-6-1523

[B30] van den Worm SH Koning RI Warmenhoven HJ Koerten HK van Duin J Cryo electron microscopy reconstructions of the Leviviridae unveil the densest icosahedral RNA packing possible J Mol Biol 2006 363 858 865 10.1016/j.jmb.2006.08.053 16989861

[B31] Kozlovska TM Cielens I Dreilinna D Dislers A Baumanis V Ose V Pumpens P Recombinant RNA phage Q beta capsid particles synthesized and self-assembled in Escherichia coli Gene 1993 137 133 137 10.1016/0378-1119(93)90261-Z 7506687

[B32] Peabody DS Translational repression by bacteriophage MS2 coat protein expressed from a plasmid. A system for genetic analysis of a protein-RNA interaction J Biol Chem 1990 265 5684 5689 2108146

[B33] Tissot AC Renhofa R Schmitz N Cielens I Meijerink E Ose V Jennings GT Saudan P Pumpens P Bachmann MF Versatile Virus-Like Particle Carrier for Epitope Based Vaccines PLOS One 2010 5 3 e9809 10.1371/journal.pone.0009809 20352110 PMC2843720

[B34] Martina BEE van den Doel P Koraka P van Amerongen G Spohn G Haagmans BL Fouchier RAM Osterhaus ADME Rimmelzwaan GF A recombinant influenza A expressing domain III of West Nile virus induces protective immune responses against influenza and West Nile virus PLOS One in press 10.1371/journal.pone.0018995 PMC3082541 21541326

[B35] Huang CY Silengo SJ Whiteman MC Kinney RM Chimeric dengue 2 PDK-53/West Nile NY99 viruses retain the phenotypic attenuation markers of the candidate PDK-53 vaccine virus and protect mice against lethal challenge with West Nile virus J Virol 2005 79 7300 7310 10.1128/JVI.79.12.7300-7310.2005 15919884 PMC1143654

[B36] Diamond MS Pierson TC Fremont DH The structural immunology of antibody protection against West Nile virus Immunol rev 2008 225 212 225 10.1111/j.1600-065X.2008.00676.x 18837784 PMC2646609

[B37] Bachmann MF Rohrer UH Kundig TM Burki K Hengartner H Zinkernagel RM The influence of antigen organization on B cell responsiveness Science 1993 262 1448 1451 10.1126/science.8248784 8248784

[B38] Spohn G Bachmann MF Exploiting viral properties for the rational design of modern vaccines Expert Rev Vaccines 2008 7 43 54 10.1586/14760584.7.1.43 18251693

[B39] Jennings GT Bachmann MF The coming of age of virus-like particle vaccines Biol Chem 2008 389 521 536 10.1515/BC.2008.064 18953718

[B40] Bachmann MF Spohn G WO2007/039552 A1: Interleukin-1 conjugates and uses thereof 2007

